# Supplementary material for: GC-MS- and NMR-Based Metabolomics and Molecular Docking Reveal the Potential Alpha-Glucosidase Inhibitors from Psychotria malayana Jack Leaves
Source: Pharmaceuticals (Basel). 2021 Sep 26;14(10):978. doi: 10.3390/ph14100978 (PMC8541227; doi:10.3390/ph14100978)
Supplement: Supplementary file 1 [file pharmaceuticals-14-00978-s001.zip › Table S1.pdf]

Table S1. Data resulted from docking of the putative bioactive compounds in alpha-glucosidase (3A4A).

Compound	Interacting amino acid residues	Bond type	Bond distance (Å)
ADG	ASP352	Hydrogen bond	2.44
	GLH277	Hydrogen bond	2.74
	ASH215	Hydrogen bond	2.43, 2.74
	HIE112	Hydrogen bond	2.18
	ASH69	Hydrogen bond	1.75, 2.12
	ARG442	Hydrogen bond	1.87
	HIE351	Hydrogen bond	1.96
Quercetin	ASH215	Hydrogen bond	2.49
	GLH277	Hydrogen bond	2.01
	PHE303	Pi-Pi T-shaped	4.97, 5.14
	ARG442	Pi-cation	3.77
	ASP352	Pi-anion	4.30
Compound **1**	ASP352	Hydrogen bond	2.35
	HIE351	Hydrogen bond	2.40
	GLN182	Hydrogen bond	2.70
	AGR442	Pi-cation	4.18
	VAL216	Pi-alkyl	5.47
Compound **2**	HIE351	Hydrogen bond	2.20
	ASH215	Hydrogen bond	2.30
	ARG442	Hydrogen bond	2.89
	PHE303	Pi-alkyl	4.66; 5.21
	TYR158	Pi-alkyl	4.91
Compound **3**	SER 311	Hydrogen bond	2.20
	TYR 72	Pi-sigma<br>Pi-alkyl	3.88<br>4.61
	PHE 178	Pi-sigma<br>Pi-alkyl	3.67<br>4.68
	TYR 158	Pi-alkyl	5.26
	HIE 280	Pi-alkyl	5.25
	PHE 303	Pi-alkyl	4.66
	ARG 315	Alkyl	4.12; 4.69

Table S1. Data resulted from docking of the putative bioactive compounds in alpha-glucosidase (3A4A) (cont).

Compound	Interacting amino acid residues	Bond type	Bond distance (Å)
Compound **4**	ARG 213	Hydrogen bond	2.89
	GLH 277	Hydrogen bond	2.90
	ASP 352	Hydrogen bond	2.87
	GLN 279	Hydrogen bond	2.92
	ARG 315	Alkyl	4.54
	PHE 303	Pi-alkyl	5.18
	HIE 280	Pi-alkyl	5.17
	TYR 158	Pi-alkyl	4.79; 5.21
Compound **5**	PHE 303	Pi-sigma	3.98
		Pi-alkyl	5.36; 5.00; 4.77
	PHE 159	Pi-alkyl	4.75
	TYR 158	Pi-alkyl	5.01
	TYR 72	Pi-sigma	3.97
	PHE 178	Pi-sigma	3.86
		Pi-alkyl	4.23; 5.35
	HIE280	Pi-alkyl	4.72
	ARG 315	Pi-alkyl	4.29
		Alkyl	4.16
Compound **6**	PRO 312	Hydrogen bond	2.92
	PHE 178	Pi-alkyl	4.56
	PHE 303	Pi-sigma	3.87
		Pi-alkyl	5.07; 4.98
	VAL 216	Alkyl	4.77
	HIE 351	Pi-alkyl	5.17
	TYR 72	Pi-sigma	3.78; 3.38
		Pi-alkyl	4.01
	HIE 280	Pi-alkyl	4.79; 4.38
	ARG 315	Pi-sigma	2.97
		Alkyl	4.15; 4.76
Compound **7**	PHE 178	Pi-sigma	3.76
		Pi-alkyl	4.65
	TYR 72	Pi-alkyl	4.85
	PHE 303	Pi-alkyl	4.13
	ARG 315	Alkyl	4.68; 4.46
	TYR 158	Pi-alkyl	4.05; 5.08

Table S1. Data resulted from docking of the putative bioactive compounds in alpha-glucosidase (3A4A) (cont).

Compound	Interacting amino acid residues	Bond type	Bond distance (Å)
Compound **8**	TYR 72	Pi-sigma	3.80; 3.82
	PHE 178	Pi-sigma	3.71
		Pi-alkyl	4.05
	PHE 303	Pi-alkyl	5.46; 4.62
	TYR 158	Pi-alkyl	5.33; 4.35
	ARG 315	Alkyl	5.03; 4.19; 4.46
	HIE 280	Pi-alkyl	4.93
	HIE 351	Pi-alkyl	5.08
	PHE 159	Pi-alkyl	5.43
Compound **10**	ARG442	Hydrogen bond	2.19
		Pi-Cation	4.69
	HIE351	Hydrogen bond	2.14
	ASH215	Hydrogen bond	2.51
	ASP352	Pi-Anion	4.95
Compound **11**	ASH215	Hydrogen bond	2.22
	ARG442	Hydrogen bond	2.18
	ASP352	Hydrogen bond	2.67; 2.73
	GLH277	Hydrogen bond	2.12; 2.46
	HIE351	Hydrogen bond	1.96
