# Supplementary figures and images for: GC-MS- and NMR-Based Metabolomics and Molecular Docking Reveal the Potential Alpha-Glucosidase Inhibitors from Psychotria malayana Jack Leaves
Source: Pharmaceuticals (Basel). 2021 Sep 26;14(10):978. doi: 10.3390/ph14100978 (PMC8541227; doi:10.3390/ph14100978)

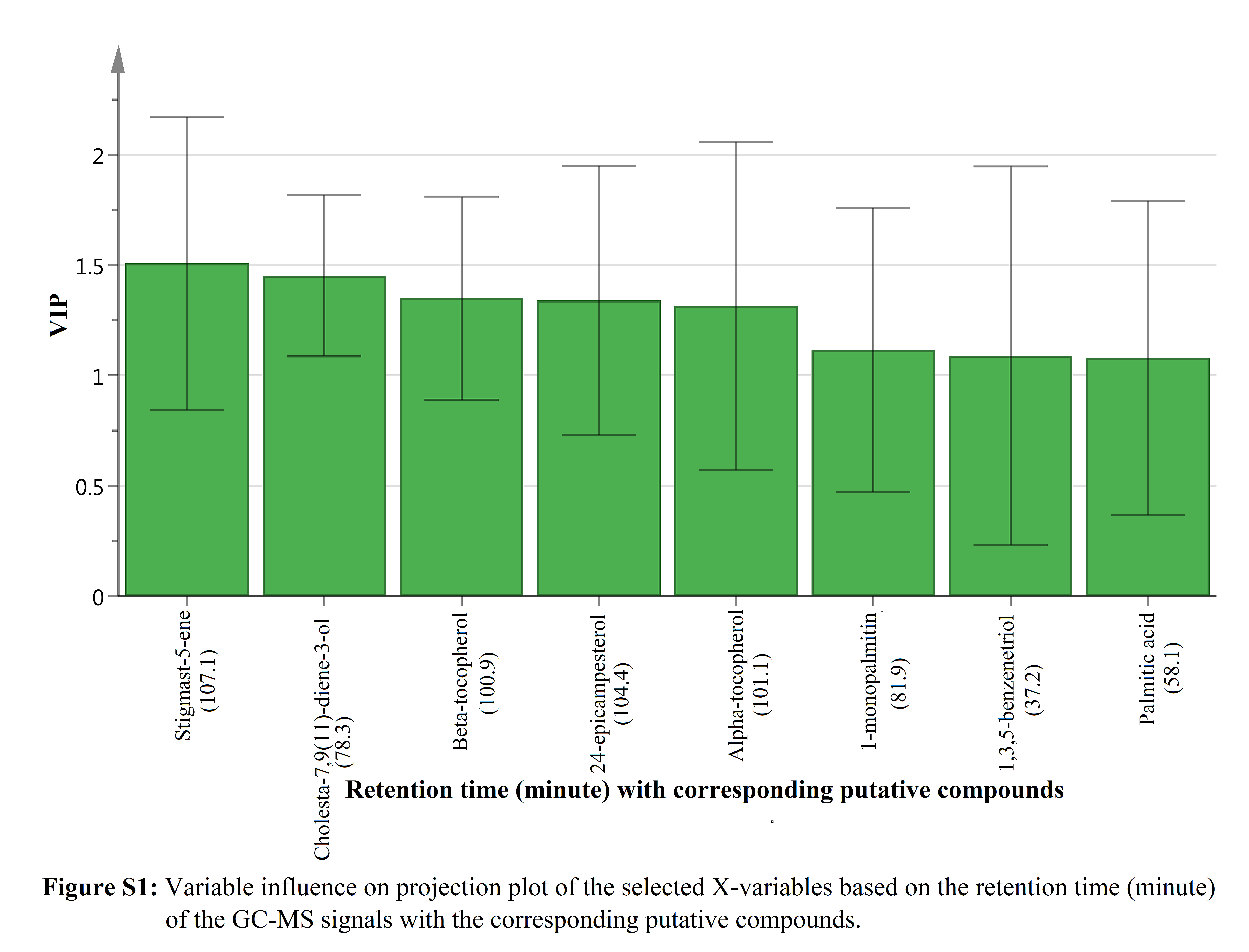

Supplement: Supplementary file 1 [file pharmaceuticals-14-00978-s001.zip › Figure S1.tiff]

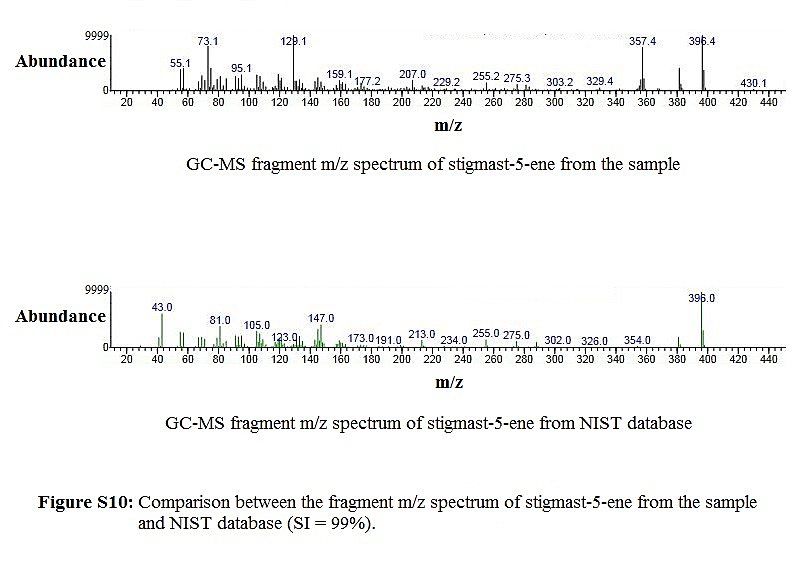

Supplement: Supplementary file 1 [file pharmaceuticals-14-00978-s001.zip › Figure S10.tiff]

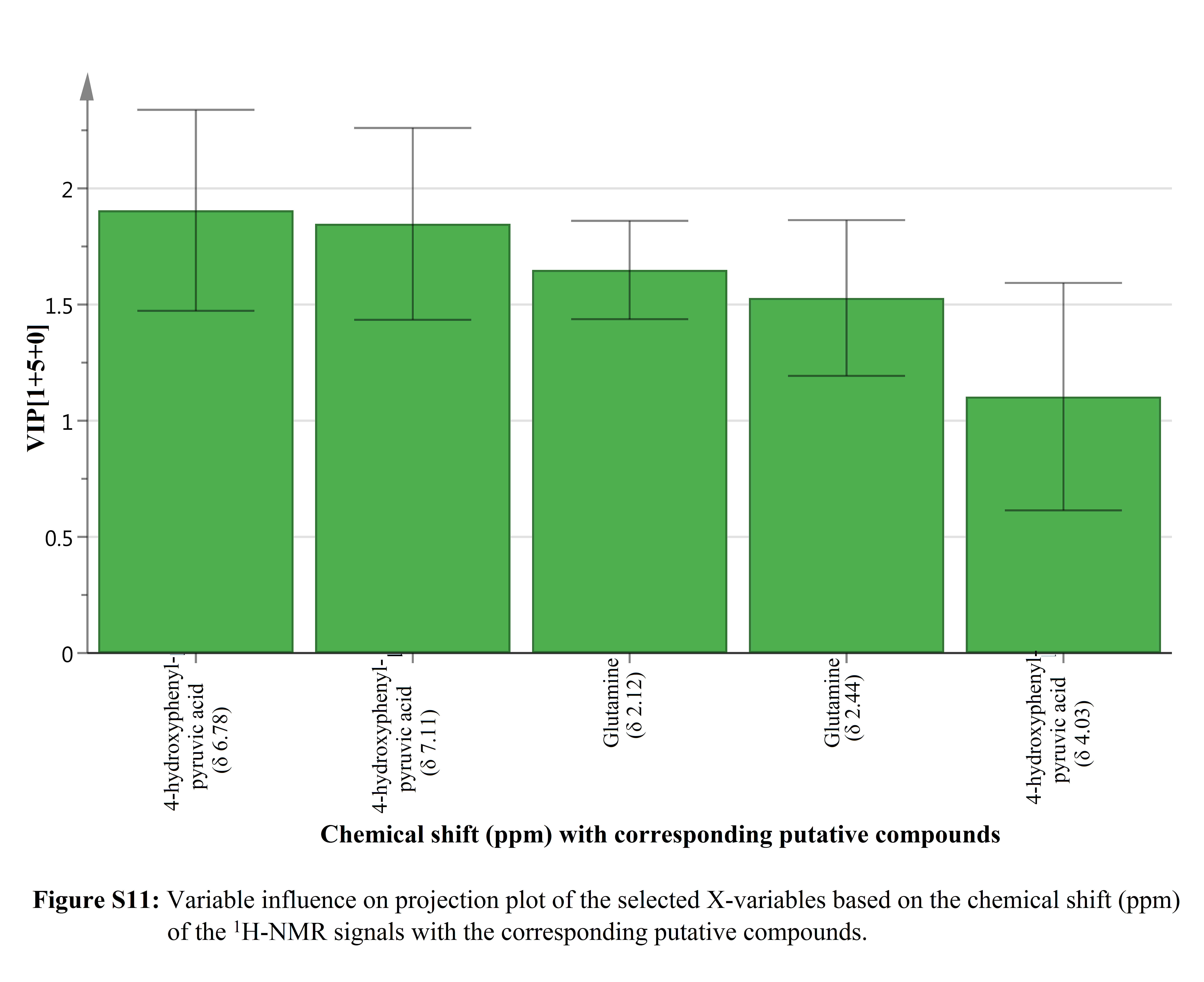

Supplement: Supplementary file 1 [file pharmaceuticals-14-00978-s001.zip › Figure S11.tiff]

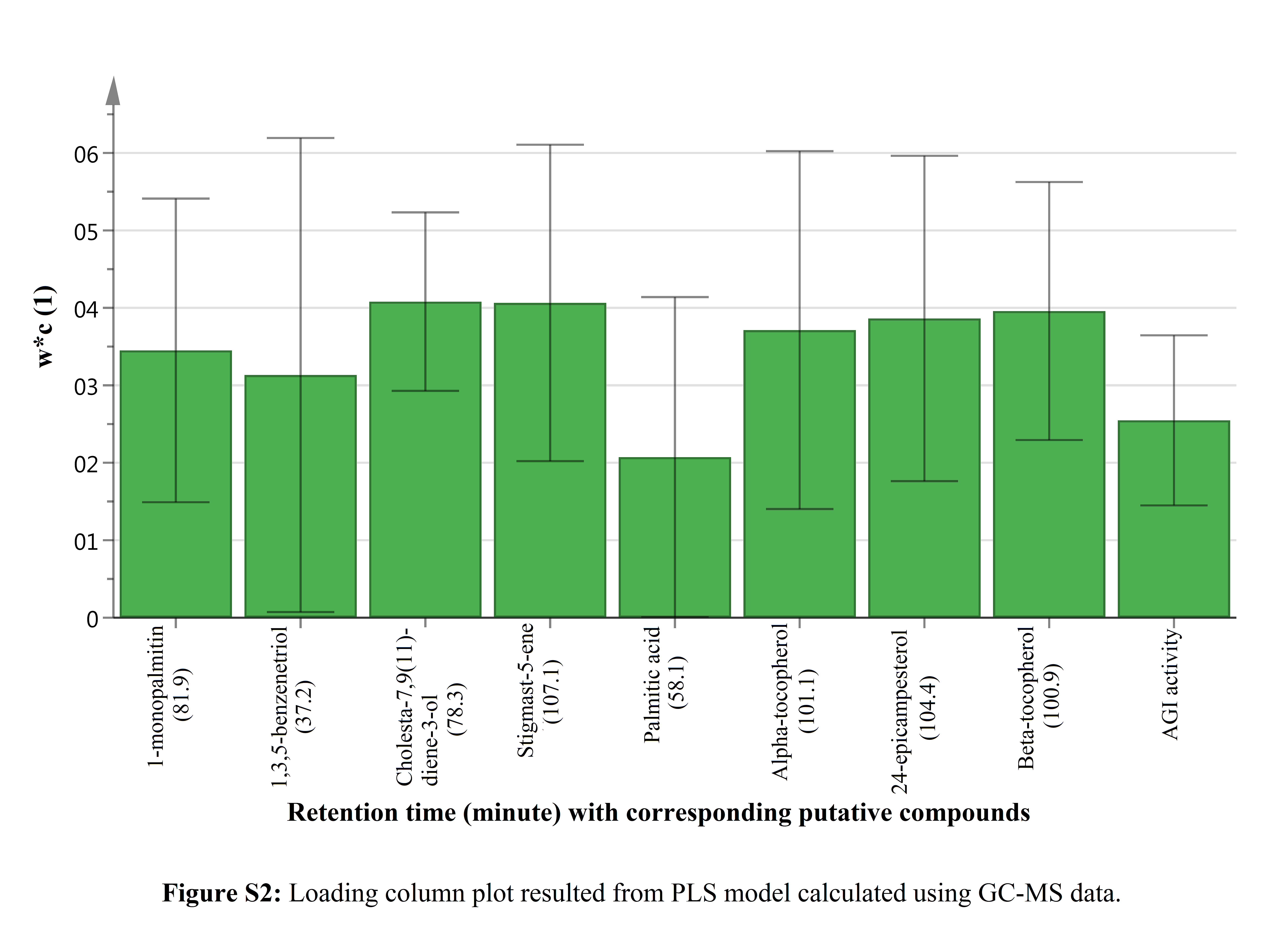

Supplement: Supplementary file 1 [file pharmaceuticals-14-00978-s001.zip › Figure S2.tiff]

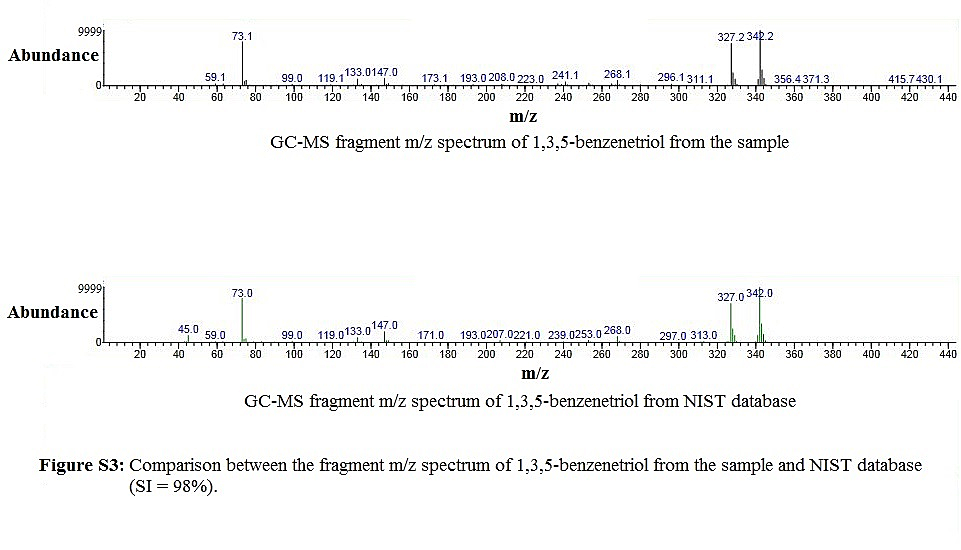

Supplement: Supplementary file 1 [file pharmaceuticals-14-00978-s001.zip › Figure S3.tiff]

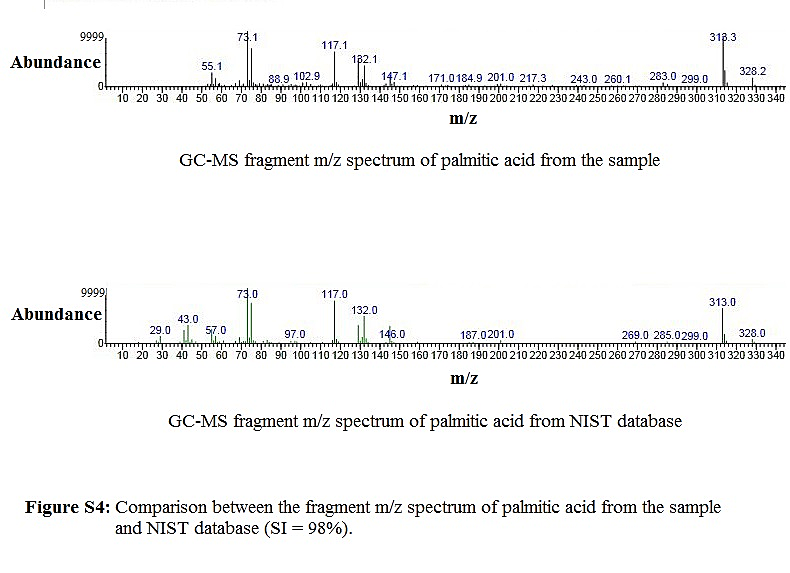

Supplement: Supplementary file 1 [file pharmaceuticals-14-00978-s001.zip › Figure S4.tiff]

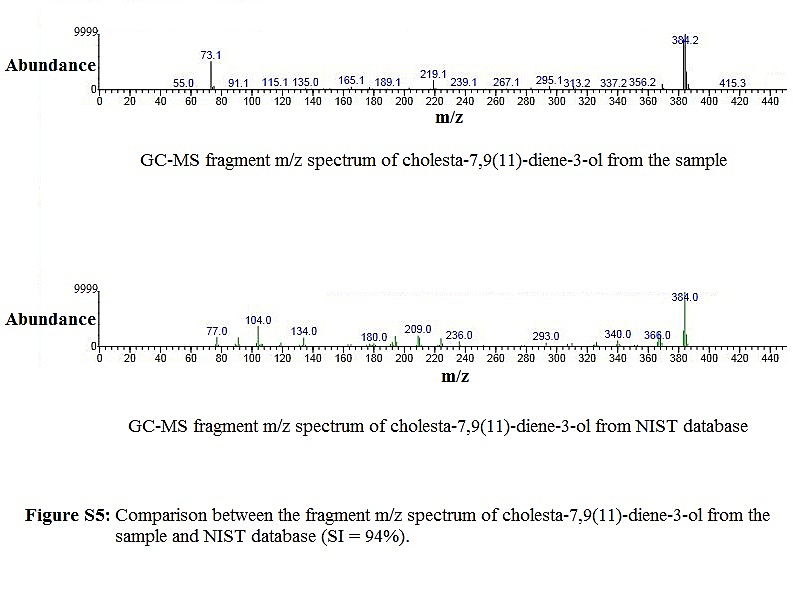

Supplement: Supplementary file 1 [file pharmaceuticals-14-00978-s001.zip › Figure S5.tiff]

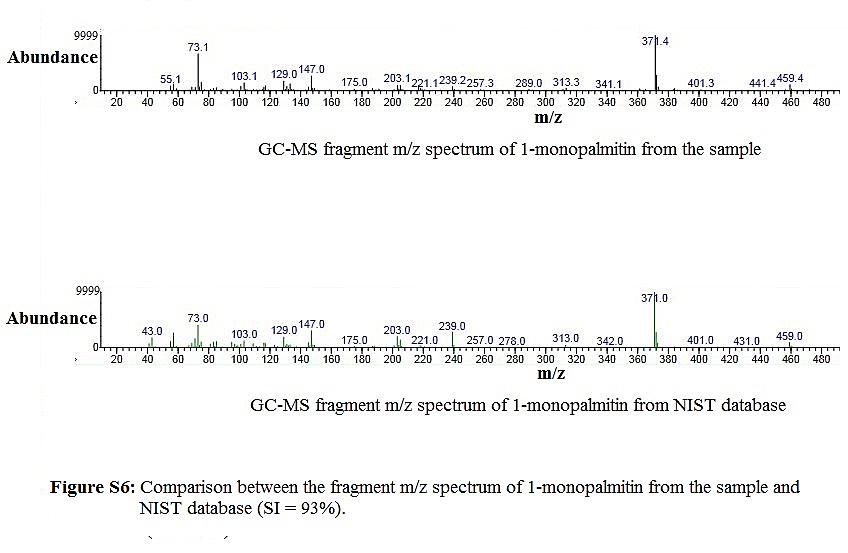

Supplement: Supplementary file 1 [file pharmaceuticals-14-00978-s001.zip › Figure S6.tiff]

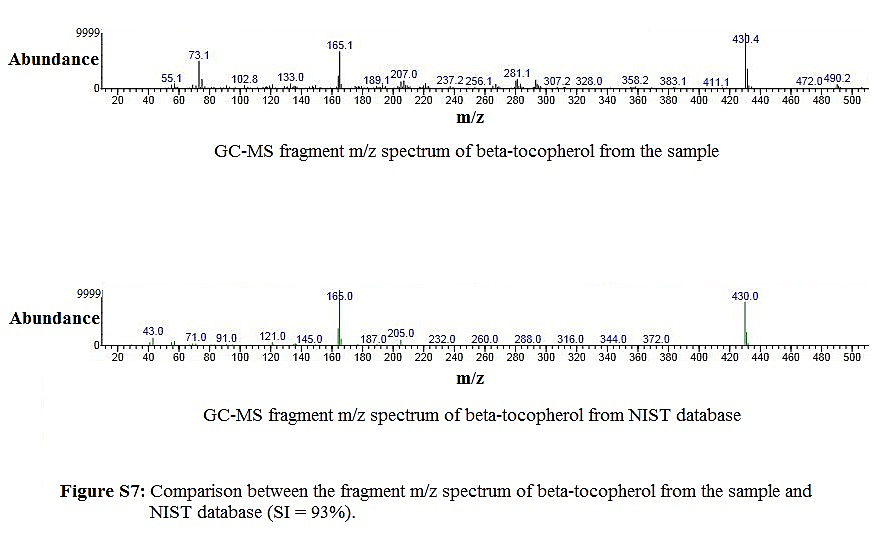

Supplement: Supplementary file 1 [file pharmaceuticals-14-00978-s001.zip › Figure S7.tiff]

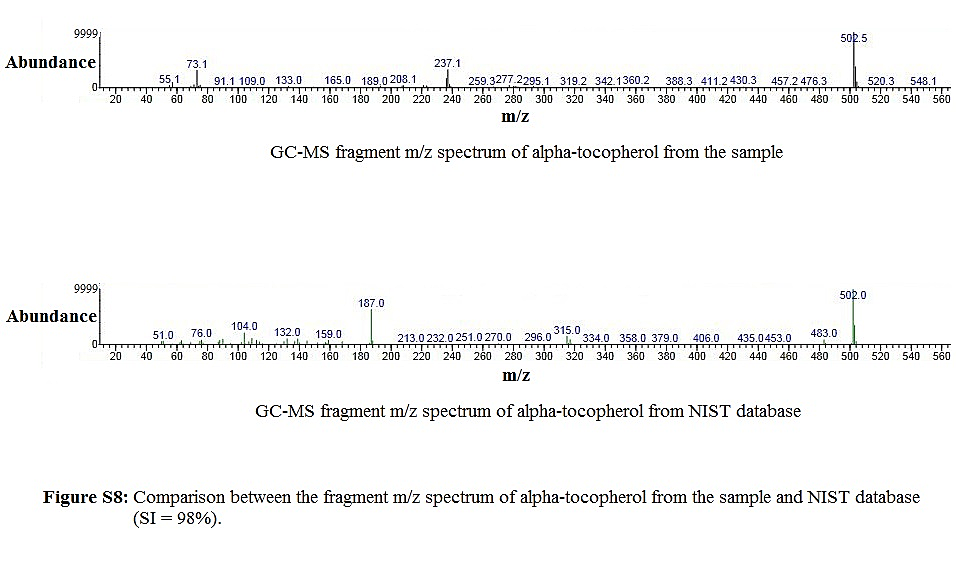

Supplement: Supplementary file 1 [file pharmaceuticals-14-00978-s001.zip › Figure S8.tiff]

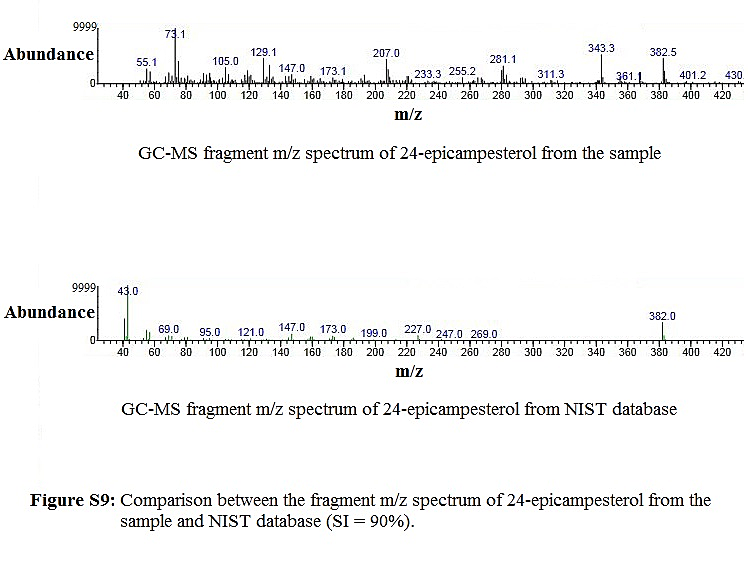

Supplement: Supplementary file 1 [file pharmaceuticals-14-00978-s001.zip › Figure S9.tiff]
